# Ultralight Graphene/Carbon Nanotubes Aerogels with Compressibility and Oil Absorption Properties

**DOI:** 10.3390/ma11040641

**Published:** 2018-04-22

**Authors:** Da Zhao, Li Yu, Dongxu Liu

**Affiliations:** 1 School of Aeronautic Science and Engineering, Beihang University, Beijing 100191, China; zhaoda@buaa.edu.cn; 2 Beijing Infinite Space Technology Co.Ltd, Beijing 100191, China; yuli101020208@163.com

**Keywords:** graphene/carbon nanotubes aerogels, ascorbic acid, compressibility, oil absorption capacity

## Abstract

Graphene aerogels have many advantages, such as low density, high elasticity and strong adsorption. They are considered to be widely applicable in many fields. At present, the most valuable research area aims to find a convenient and effective way to prepare graphene aerogels with excellent properties. In this work graphene/carbon nanotube aerogels are prepared through hydrothermal reduction, freeze-drying and high temperature heat treatment with the blending of graphene oxide and carbon nanotubes. A new reducing agent-ascorbic acid is selected to explore the best preparation process. The prepared aerogels have compression and resilience and oil absorption properties due to the addition of carbon nanotubes as designed.

## 1. Introduction

Graphene has excellent optical, electrical, thermal and mechanical properties and high surface area but these properties are based on monolithic graphite. To take advantage of these properties, the monolayer graphene must be assembled into macroscopic materials, such as graphene films, graphene fibers and 3D graphene aerogels (GAs). GAs have attracted wide attention from scientists due to their ultra-low density blow (10 mg/cm^3^) [1], high porosity [2] and mechanical stability [3]. GAs have wide application prospect in many fields [4,5,6,7,8], including supercapacitors, catalysis, sensors, actuators, damping materials, thermal insulation, and environmental remediation.

However, GAs are formed by self-assembly processes, which are mainly affected by Van der Waals forces and π–π bonds [9] between layers of the graphene sheet. Therefore, the elastic energy storage of GA is low [10], and GAs even can’t bear a larger force. Meanwhile, in order to prepare GAs with a lower density, the concentration of the graphene oxide solution needs to be reduced. It is easy to cause a serious shrinkage of graphene hydrogel volume in the preparation process and thus a degradation of the mechanical properties of the GA. In most cases, GAs need low density and excellent mechanical and elastic properties at the same time. Recently, lots of work by various methods [11,12,13] has been done to improve the elasticity and strength of GAs by improving the properties of graphene oxide (GO), exploring new preparation processes for aerogels and doping with other organic or inorganic compounds.

Carbon nanotubes (CNTs) have good mechanical properties [14,15] and interesting electronic properties [16,17]. The tensile strength of CNTs can reach 50–200 GPa, which is 100 times that of steel. However, their density is only 1/6 that of steel, which is at least one order of magnitude higher than that of conventional graphite fiber. The elastic modulus of CNTs can reach 1 TPa, which is equivalent to the elastic modulus of diamond and about five times that of steel. For single walled CNTs with ideal structure, the tensile strength is about 800 GPa. CNTs are the materials with the highest specific strength at present. Therefore, CNTs have widely application prospects for improving the properties of composite materials [18] and monolithic carbon aerogel nanocomposites [19].

If CNTs can be added to GA, CNTs and graphene can play a synergistic role through their π–π bonds [20]. Large layers of graphene are curled, deformed, stacked up, and then overlap to form a frame structure. The CNTs disperse uniformly on the surface and junction points of graphene which can enhance the stability of the frame structure [21]. Therefore, the addition of CNTs increases the elasticity and strength of aerogels.

In this paper, the two aspects are focused on: (1) CNTs are mixed in the solution of GO so that the aerogels prepared are homogeneous mixtures containing graphene and CNTs; (2) Ascorbic acid is chosen as a reducing agent to make the hydrothermal reduction reaction of GO take place under mild reaction conditions. The lamellar graphene layers are close to each other, and the hydrogel is formed on the macro surface. The water solvent is removed from the hydrogel to become an aerogel through a freeze-drying process. Then the structure and properties of graphene/CNTs aerogels are provided. Test results are consistent with the design results.

## 2. Materials and Methods

**Figure 1:**
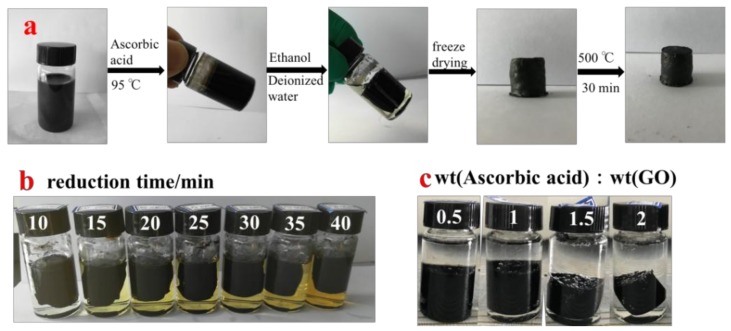
(**a**) Illustration of the synthesis process of graphene/CNTs ; (**b**) different reduction time; (**c**) different mass ratio of ascorbic acid and graphene oxide.

The preparation process of graphene/CNT aerogels is shown in Figure 1a. Firstly, a certain amount of GO solution (2–10 mg/mL) and CNTs are mixed under ultrasonic irradiation for 1 h, which makes the GO and CNTs disperse evenly. Then a certain amount of ascorbic acid (ascorbic acid:GO(wt) = 0.5, 1, 1.5, 2) is added into the mixed solution as the GO reducing agent. The mixed solution is exposed to ultrasoound for 5 min to ensure that ascorbic acid is completely dissolved and dispersed evenly and then the mixture is heated in an oil bath at 95 °C. The reduction reaction of GO occurs under the effect of the heat and ascorbic acid. Due to the reduction of GO, the color of the solution firstly changed from dark brown to black. The layers become close to each other to form a hydrogel with a slight volume contraction. The degree of volume shrinkage has a great influence on the density of aerogels. Therefore, in the synthesis process of graphene/CNTs aerogels, the effects of reaction time and amount of ascorbic acid on the volume shrinkage of gel were investigated. Results are illustrated in Figure 1b,c.

With the increase of hydrothermal reaction time, the volume shrinkage of the hydrogel becomes more and more serious. With the increase of ascorbic acid amount, the volume shrinkage is also more and more serious. Then graphene/CNTs hydrogel is dialysed for 24 h using ethanol/deionized water solution in order to prevent the frost heave phenomenon in the subsequent freeze-drying process which will make the aerogels crack and destroy the macrostructure integrity of the aerogels. Such a destruction would inevitably lead to a decline of the mechanical properties of the aerogels. Therefore, the dialysis process for graphene/CNTs hydrogels is a key step in the whole preparation process. After dialysis, the freeze-drying is used to remove water to guarantee that there is no volume reduction, structural deformation or collapse during drying. Then the aerogels are heated at 500 °C under an argon atmosphere. Through the process of heat treatment, the graphene is further reduced so that the six element ring structure is further repaired, and the superfluous ascorbic acid is decomposed at the same time. Finally, the pure reduced graphene/CNTs aerogels are obtained.

## 3. Results

### 3.1. The Density of Grapheme/CNTs Aerogels

**Figure 2:**
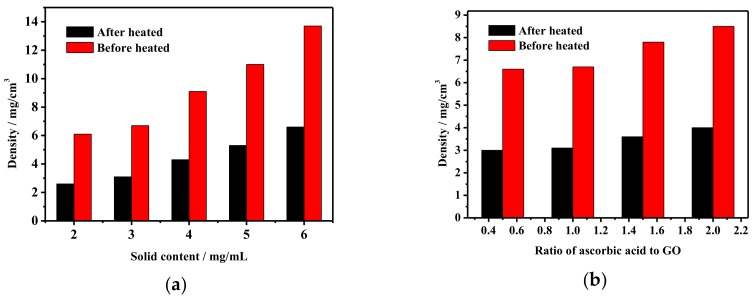
The density of aerogels: (**a**) Aerogel density before and after heat treatment; (**b**) Aerogel density under different ascorbic acid content.

The density of aerogels has a very important impact on their properties and applications. Therefore, this paper explores the factors that influence the aerogel density during the preparation process. Firstly, the influence of final heat treatment on aerogel density during the preparation process is discussed and the result is illustrated in Figure 2a. Taking solid content is 3 mg/mL as an example, the density of aerogels before heat treatment is 7 mg/cm^3^ and the density of aerogels after heat treatment is 3 mg/cm^3^. The density of aerogels after heat treatment is reduced by more than half compared with that before heat treatment. This is because the heat treatment makes the residual ascorbic acid decompose and some functional groups of the reduced graphene can be degraded at the same time. Therefore, to a great extent, the quality of aerogels is reduced. The pore structure of the aerogels is uniform and well-arranged under the compression of ice crystal structure during the freezing process. Therefore, the perfect 3D pore structure of aerogels with the joint action of the graphene and CNTs can withstand the high temperature without volume reduction or structural deformation. Therefore, the heat treatment can greatly reduce the density of graphene/CNTs aerogels.

In addition, as for the preparation process, the smaller the volume shrinkage during the hydrothermal reduction step, the greater density of the resulting aerogels. Therefore, we need to find a balance point that can form a complete hydrogel and guarantee a slight volume contraction. By controlling the reaction time and the dosage of ascorbic acid, the optimum reduction degree is finally found. The effect of ascorbic acid on aerogel density during preparation of graphene/CNTs aerogels is investigated and the result is shown in Figure 2b. When the mass ratio of ascorbic acid to GO is increased from 0.5 to 2, the density before heat treatment increases from 6.6 mg/cm^3^ to 8.5 mg/cm^3^, and the density after heat treatment increases from 3.0 mg/cm^3^ to 4.0 mg/cm^3^. The reason is that the increase of the reducing agent leads to the rate and degree of the hydrothermal reduction reaction increase and the force between the lamellae of graphene is increased, therefore, the porosity and pore size of hydrogel and aerogel and the density of the aerogel increases.

### 3.2. Macrostructure and Microstructure of Graphene/CNTs Aerogels

**Figure 3:**
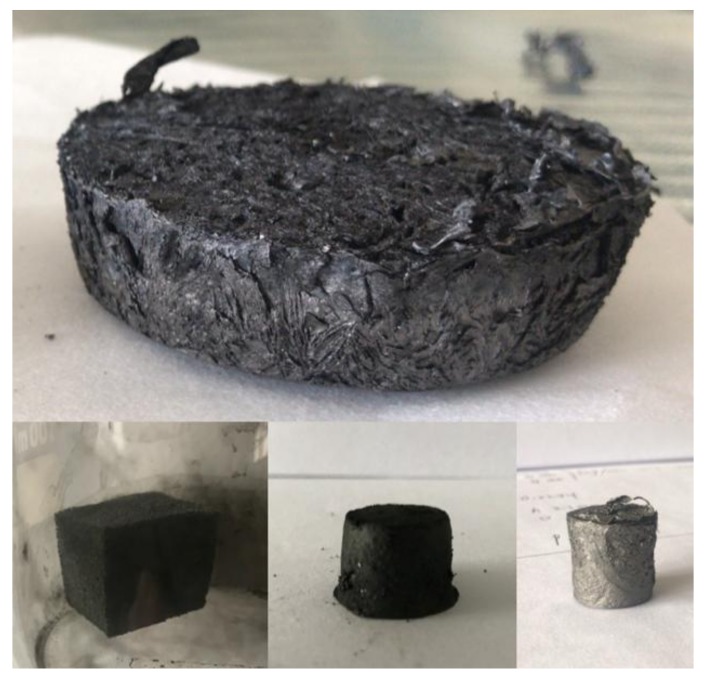
The macrostructure of graphene/CNTs aerogels.

The macrostructure of graphene/CNTs aerogels is shown in Figure 3. The graphene/CNTs aerogels have macro continuity and integrity. Therefore, aerogels can be designed to different shapes and sizes according to their needs. Moreover, with the increase of carbon nanotube content, the color of aerogels is getting darker and darker. Therefore, the content of carbon nanotubes in aerogels can be judged simply by color.

**Figure 4:**
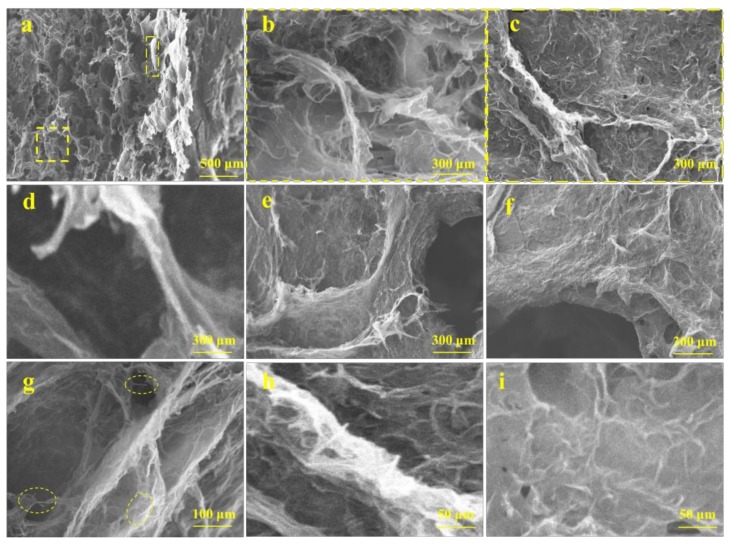
SEM image of the microstructure of graphene/CNTs aerogels; (**a–c**) SEM image of cross-section structure of graphene/CNTs aerogels under different magnification; (**d**) SEM image of the morphologies of curly graphene; (**e**) SEM image of the morphology of distorted graphene; (**f**) SEM image of the morphology of stacked graphene; (**g**) SEM image of the morphology of CNTs at different positions; (**h**) SEM image of the morphology of CNTs at the crosslinking point; (**i**) SEM image of the morphology of CNTs on the surface of graphene.

The microstructure of graphene/CNTs aerogels was observed by scanning electron microscope (SEM) and the results are shown in Figure 4. As seen in Figure 4a–c, there are many pore structures in the aerogels, and the size of the micropores varies from several microns to several nanometers. This is consistent with the structure and size of the pure graphene aerogels. The walls of pores are made up of a large amount of graphene. During the freeze drying process, the graphene is rearranged along the neat ice crystal structure, and finally forms a large size three-dimensional pore structure with uniform arrangement and uniform size. Regular void structure helps to improve the mechanical properties of aerogels. Figure 4d–f show the morphology of the walls of the pores. It can be seen that large scale graphene exhibits a phenomenon of twisting, bending, and stacking. Layered graphene interlinking and crosslinking at the edge of the lamellar under the action of chemical reaction and carbon nanotubes. Figure 4g–i show that CNTs are intertwined and uniformly dispersed on the surface of graphene, the curled inner wall and the lamellar structure connection, which can improve the stability of graphene structural units effectively and store a certain amount of energy. Then, the porous structure formed by graphene and CNTs is formed as a basic unit, which is connected to other unints in a connected three-dimensional network macrostructure by interconnected stacking.

### 3.3. Compressibility and Influencing Factors of Graphene/CNTs Aerogels

**Figure 5:**
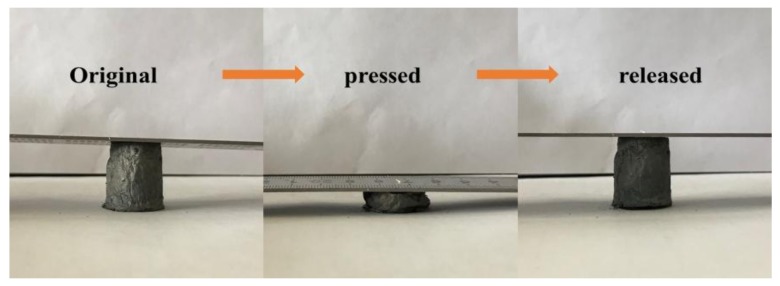
Compression and resilience of graphene/CNTs.

The prepared aerogels have excellent compression and resilience due to the introduction of CNTs, as shown in Figure 5. Taking the aerogel with 50% CNTs content as an example. When the aerogel is compressed to its own height of 90%, it can still quickly rebound to its original height without structural collapse or permanent deformation. These aerogels exhibits excellent compression resilience compared with pure graphene aerogels. At the same time, similar to other porous materials, there is a negative Poisson's ratio in vertical direction compression.

During the compression process, the pore network structure of pure graphene aerogels is extruded into almost dense parallel structures. The storage energy is very low. From the macro structure viewpoint, there is an unrecoverable destruction. In the structure of graphene/CNTs aerogels, graphene is wrapped by CNTs wrapped around each other. There is a synergistic effect between graphene and CNTs during the compression process. Therefore, the external force can be transferred to the carbon nanotube through the graphene. In the process of compression, the slip of the graphene layer and the fracture of the connection point can be avoided by CNTs so that the porous network structure is very stable. Meanwhile, CNTs also store a lot of energy to ensure that the aerogels can restore the original shape when the external force is removed.

**Figure 6:**
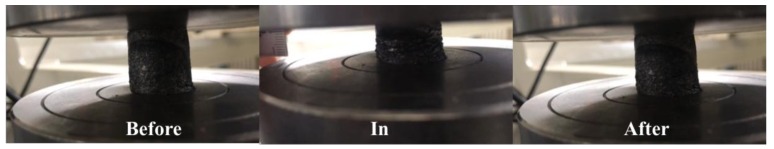
Macrostructure of before, in and after compression.

**Figure 7:**
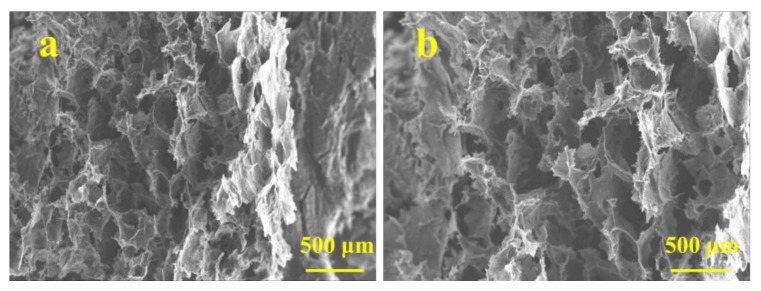
(**a**) SEM image of cross-section structure of graphene/CNTs aerogels; (**b**) SEM image of cross-section structure of graphene/CNTs aerogels after compression for 10 times.

Taking the aerogel with density of 5 mg/cm^3^ as an example. In order to verify the stability of the elastic properties of aerogels, the compression fatigue tests of 1, 5 and 10 times are repeated at the compression speed of 10 mm/min. As is shown in Figure 6, the macrostructure of aerogels has not been changed significantly after compression for 10 times, and the volume don’t decrease and no cracks appeared. Figure 7 shows that there is no obvious difference in microstructure before and after compression for 10 times, and aerogels still maintain a stable structural unit of graphene and CNTs. From the above macroscopic and microscopic structure tests, it can be concluded that graphene/CNTs aerogel has excellent elastic properties for a long time.

**Figure 8:**
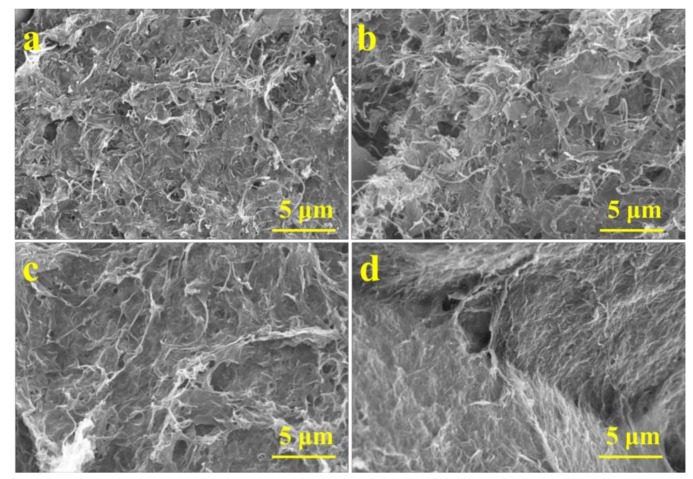
SEM image of aerogels with with different CNTs content; (**a**) GO:CNTs(wt) = 1:2; (**b**) GO:CNTs(wt) = 1:1; (**c**) GO:CNTs(wt) = 2:1; (**d**) GO:CNTs(wt) = 3:1.

In order to study the influencing factors of the elastic properties of graphene/CNTs aerogels, taking the aerogel with density of 5 mg/cm^3^ as an example. The maximum recoverable elastic deformation of aerogels with different CNTs content is investigated, and the result is shown in Table 1. With the increase of CNT content, the elastic properties are getting better and better. Until the CNTs content reaches 50%, the performance of aerogels is optimal, and the recoverable compression deformation reaches 90% and then with the increase of CNTs, the elastic properties decrease gradually. When the CNTs content reaches 90%, the recoverable compression deformation is only 45%. Figure 8a–d show the micromorphology of aerogels with different CNT contents. When the content of CNTs is 10%, CNTs are evenly distributed on the surface of the graphene layer, and the tubes can’t be closely connected. Therefore, the structure of the graphene can’t be greatly supported by CNTs and the elastic properties of the aerogels are only slightly increased. With the content of CNTs increased to 50%, CNTs form a dense network structure and are intertwined with each other on the surface of graphene so that they could absorb and store large amount of energy transferred by graphene and aerogels have the best elastic performance. With the further increase of CNTs content, the CNTs are reunited to some extent, which destroys the stable pore network structure. So the elastic properties decrease gradually. It can be concluded that the elastic properties of aerogels are greatly influenced by the content of CNTs.

**Figure 9:**
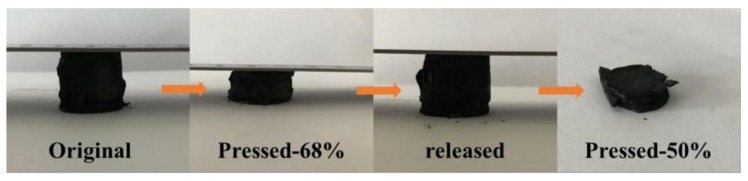
Recoverable deformation of aerogels without heat treatment.

The excellent elastic properties of aerogels are due to the synergistic effect of graphene and CNTs. Therefore, in addition to the influence of the content of CNTs, the interaction force between graphene and CNTs also has a great affect. The greater the binding force between graphene and CNTs, the better this synergistic effect is, and the better the elastic properties of the aerogel. It can be seen from Figure 9 that the recoverable deformation of aerogels without heat treatment is 68%. After more than 50% deformation, the structure collapsed and could not be restored to its original state, so the elastic properties are much less than the aerogels treated by high temperature heat treatment. This is due to the removal of the superfluous ascorbic acid and other impurities in the process of high temperature heat treatment. The six membered ring structure of graphene and the acidified carbon nanotubes have been repaired to a certain extent. The effect of π–π bonding between graphene and CNTs is obviously enhanced and the synergistic effect is more remarkable. Therefore, the aerogel after heat treatment under a high temperature has more excellent elastic properties.

### 3.4. Hydrophobicity and Hydrophobicity of Grapheme/CNTs Aerogels

The hydrophilic properties of the prepared aerogel are tested, and the water contact angles of aerogels with different CNTs content are shown in Table 2. Because the surface structure of the partially reduced graphene still has oxygen functional groups, its hydrophobicity is not obvious, and the water contact angle of pure graphene aerogels is about 92°. The water droplets infiltrate into aerogels after a period of time, which makes the quality of aerogels obviously increased. With the increase of CNTs content, the water contact angle increases obviously, indicating that the hydrophobicity of aerogels is obviously improved. When the CNTs content reaches 20%, the water contact angle is more than 120°, showing obvious super hydrophobicity. When the CNTs content is over 50%, its super hydrophobic performance tends to become stable.

**Figure 10:**
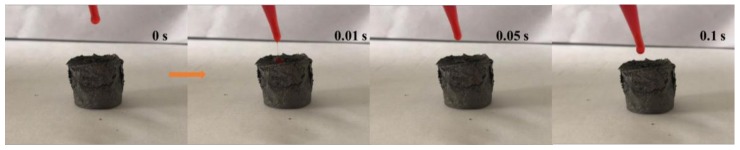
Lipophilicity of aerogels.

**Figure 11:**
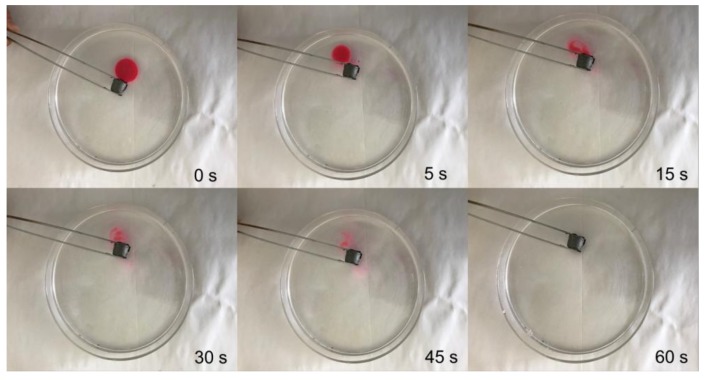
Water and oil separation performance of aerogels.

The oil affinity test of the aerogels is carried out, and the results are that aerogels showed super strong affinity to oil, as shown in Figure 10. When the oil drops fall onto the aerogel surface, the oil drops quickly penetrate into the aerogel, and the whole process takes no more than 1 s. As is shown in Figure 11, when separating oil and water mixtures, the aerogel floats on the surface of water and rapidly separates the floating organic matter, which shows its selective adsorption effect for oil and water. Therefore, the addition of carbon nanotubes can significantly enhance the hydrophobicity and lipophilicity of aerogels.

### 3.5. Oil Absorption Capacity and Influencing Factors of Grapheme/CNTs Aerogels

In order to explore the adsorption ability of aerogels to different oils, several different oil products are selected, and their basic properties are listed in Table 3. The viscosity and density of each oil product differ greatly. Therefore, the adsorption properties of aerogels on these oils represent the oil absorption property of the prepared aerogels.

**Figure 12:**
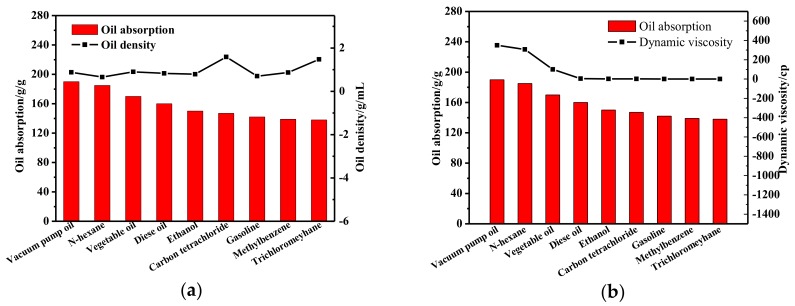
The adsorption capacity of aerogels to oils (**a**) The adsorption capacity of aerogels to oils with different density; (**b**) The adsorption capacity of aerogels to oils with different dynamic viscosity.

We take aerogels of graphene and CNTs mass ratio is 3:1 as an example to explore the oil absorption of different oil products. The result is shown in Figure 12. It can be found that the adsorption capacity is more than 100 g/g to each oil product. With the change of oil density, the adsorption amount of aerogel has not changed greatly, and with the viscosity of oil increases, the absorption of aerogels also increased, and showed a good linear relationship.

As an oil absorbing material, aerogels have good compression resilience performance. After the adsorption of oil, the oil pollution can be discharged simply by extrusion, and the shape and structure of the aerogels are not destroyed during the extrusion process. Therefore, aerogels as an oil absorbing material can be recycled, and the amount of oil contaminated by extrusion can reach 63%, and the recovery of low density oil, such as gasoline, can be over 80%. The recovery and efficiency of adsorption materials are achieved.

## 4. Discussion

In this paper, a method of preparation of graphene/CNTs aerogels is developed. Ascorbic acid is chosen as a reducing agent for hydrothermal reaction, and then aerogels undergo the process of freeze drying and heat treatment. When the mass ratio of graphene and ascorbic acid is 1:2, the volume shrinkage degree is minimum and the aerogel density is the smallest. When the CNTs content is 50%, aerogels have the best performance. The resulting graphene/CNTs aerogels show a density as 3 mg/cm^3^ so they can be considered ultralight materials. However, when the aerogel with 50% CNTs content is compressed to its own height of 90%, it can still quickly rebound to its original height without structural collapse or permanent deformation, showing excellent compressibility. The oil absorption capacity reaches 100 g/g, showing excellent oil absorption properties. Graphene/CNTs aerogels are also hydrophobic so that they can be used can be used as materials for oil and water separation.

## Figures and Tables

**Table 1:** Maximum recoverable elastic deformation of aerogels with different CNTs content.

CNTs Content (%)	Maximum Recoverable Elastic Deformation of Aaerogels (%)
0	10
5	40
10	50
30	70
50	90
70	65
90	45
100	0

**Table 2:** Water contact angles of aerogels with different CNTs content.

CNTs Content (%)	Water Contact Angles of Aerogels (°)
0	92
5	101
10	115
30	120
50	123
70	122
90	124
100	122

**Table 3:** Types and properties used in oil sorption testing.

Oil Species	Oil Density (g/mL)	Dynamic Viscosity (cP)
*n*-Hexane	0.66	307
Ethanol	0.79	1.2
Methylbenzene	0.87	0.69
Vacuum pump oil	0.88	350
Diesel oil	0.83	4.3
Gasoline	0.7	0.8
Vegetable oil	0.9	100
Trichloromethane	1.48	0.56
Carbon tetrachloride	1.59	0.97
